# IBD Subtype-Regulators *IFNG* and *GBP5* Identified by Causal Inference Drive More Intense Innate Immunity and Inflammatory Responses in CD Than Those in UC

**DOI:** 10.3389/fphar.2022.869200

**Published:** 2022-04-06

**Authors:** Sheng Gao, Yichen Li, Dingfeng Wu, Na Jiao, Li Yang, Rui Zhao, Zhifeng Xu, Wanning Chen, Xutao Lin, Sijing Cheng, Lixin Zhu, Ping Lan, Ruixin Zhu

**Affiliations:** ^1^ Department of Bioinformatics, Putuo People’s Hospital, Tongji University, Shanghai, China; ^2^ Guangdong Provincial Key Laboratory of Colorectal and Pelvic Floor Diseases, Department of Colorectal Surgery, The Sixth Affiliated Hospital, Guangdong Institute of Gastroenterology, Sun Yat-sen University, Guangzhou, China; ^3^ National Clinical Research Center for Child Health, The Children’s Hospital, Zhejiang University School of Medicine, Hangzhou, China; ^4^ State Key Laboratory of Biotherapy, West China Hospital, Sichuan University and Collaborative Innovation Center, Chengdu, China; ^5^ School of Medicine, Sun Yat-sen University, Shenzhen, China

**Keywords:** inflammatory bowel disease, Crohn’s disease, ulcerative colitis, causal inference, IBD subtype-regulator

## Abstract

**Background:** The pathological differences between Crohn’s disease (CD) and ulcerative colitis (UC) are substantial and unexplained yet. Here, we aimed to identify potential regulators that drive different pathogenesis of CD and UC by causal inference analysis of transcriptome data.

**Methods:** Kruskal–Wallis and Dunnett’s tests were performed to identify differentially expressed genes (DEGs) among CD patients, UC patients, and controls. Subsequently, differentially expressed pathways (DEPs) between CD and UC were identified and used to construct the interaction network of DEPs. Causal inference was performed to identify IBD subtype-regulators. The expression of the subtype-regulators and their downstream genes was validated by qRT-PCR with an independent cohort.

**Results:** Compared with the control group, we identified 1,352 and 2,081 DEGs in CD and UC groups, respectively. Multiple DEPs between CD and UC were closely related to inflammation-related pathways, such as NOD-like receptor signaling, IL-17 signaling, and chemokine signaling pathways. Based on the priori interaction network of DEPs, causal inference analysis identified *IFNG* and *GBP5* as IBD subtype-regulators. The results with the discovery cohort showed that the expression level of *IFNG*, *GBP5*, and *NLRP3* was significantly higher in the CD group than that in the UC group. The regulation relationships among *IFNG*, *GBP5*, and *NLRP3* were confirmed with transcriptome data from an independent cohort and validated by qRT-PCR.

**Conclusion:** Our study suggests that *IFNG* and *GBP5* were IBD subtype-regulators that trigger more intense innate immunity and inflammatory responses in CD than those in UC. Our findings reveal pathomechanical differences between CD and UC that may contribute to personalized treatment for CD and UC.

## Introduction

Inflammatory bowel disease (IBD) is a chronic, nonspecific, and inflammatory intestinal disease that causes significant morbidity to patients and an enormous burden to the society (Britzen-Laurent et al., 2016). The last decades have seen increased prevalence of IBD, especially in newly industrialized countries, whose number of cases increased from 3.8 million in 1990 to more than 6.8 million in 2017 with a growth rate of 85.1% (GBD 2017 Inflammatory Bowel Disease Collaborators, 2020; Kaplan and Ng, 2017). Though the mechanism still remains elusive, substantial investigations have revealed that multiple factors, including genetic susceptibility, intestinal epithelial barrier deficiency, immunological dysfunction, and nervous system dysregulation and the interactions among them, contribute to the pathogenesis of IBD (Knights et al., 2013).

Crohn’s disease (CD) and ulcerative colitis (UC), two major subtypes of IBD, have significantly different clinical presentations and markedly distinct disease courses. The manifestations of CD are discontinuous transmural asymmetric lesions which can affect the entire alimentary canal, while UC is characterized by the diffuse inflammation that is confined to the superficial mucosa of the colon, with possible involvement of the rectum (Bernstein et al., 2016). Currently, endoscopy is commonly used for the differential diagnosis of CD and UC, with a risk of bowel perforation (Kumar et al., 2019). Moreover, it is nowadays challenging to choose the best diagnostic test and the treatment protocol for the management of either CD or UC, which often results in unsatisfactory patient outcomes. Therefore, it is critical to understand the disease signature for each subtype in order to provide the most appropriate and personalized treatment for IBD patients. Previous studies suggested that the genetic biomarkers, namely, nucleotide-binding oligomerization domain-containing protein 2 (*NOD2*), immunity-related GTPase M (*IRGM*), and autophagy-related gene (*ATG16L1*) are specific for CD while interleukin 10 (*IL10*) and the human leukocyte antigen (*HLA*) are specific for UC (Uniken Venema et al., 2017). Importantly, susceptibility genes could cause the dysfunction of the intestinal epithelium and immune system, which leads to the invasion of gut microbiota. For instance, some studies reported that the deficiencies in susceptibility genes *NOD2* and *ATG16L1* may contribute to the IBD risk, due to the impaired anti-inflammatory responses and the further defects in microbial clearance (Chu et al., 2016). Furthermore, based on colon transcriptome, our previous study revealed that the colonic immunity of CD and UC is differently activated toward various pathogens (Yang et al., 2019). Overall, due to the complex interactions between genes and phenotypes, the driving force that causes the different IBD subtypes was still not identified, which is the goal of the current study.

**FIGURE 1:**
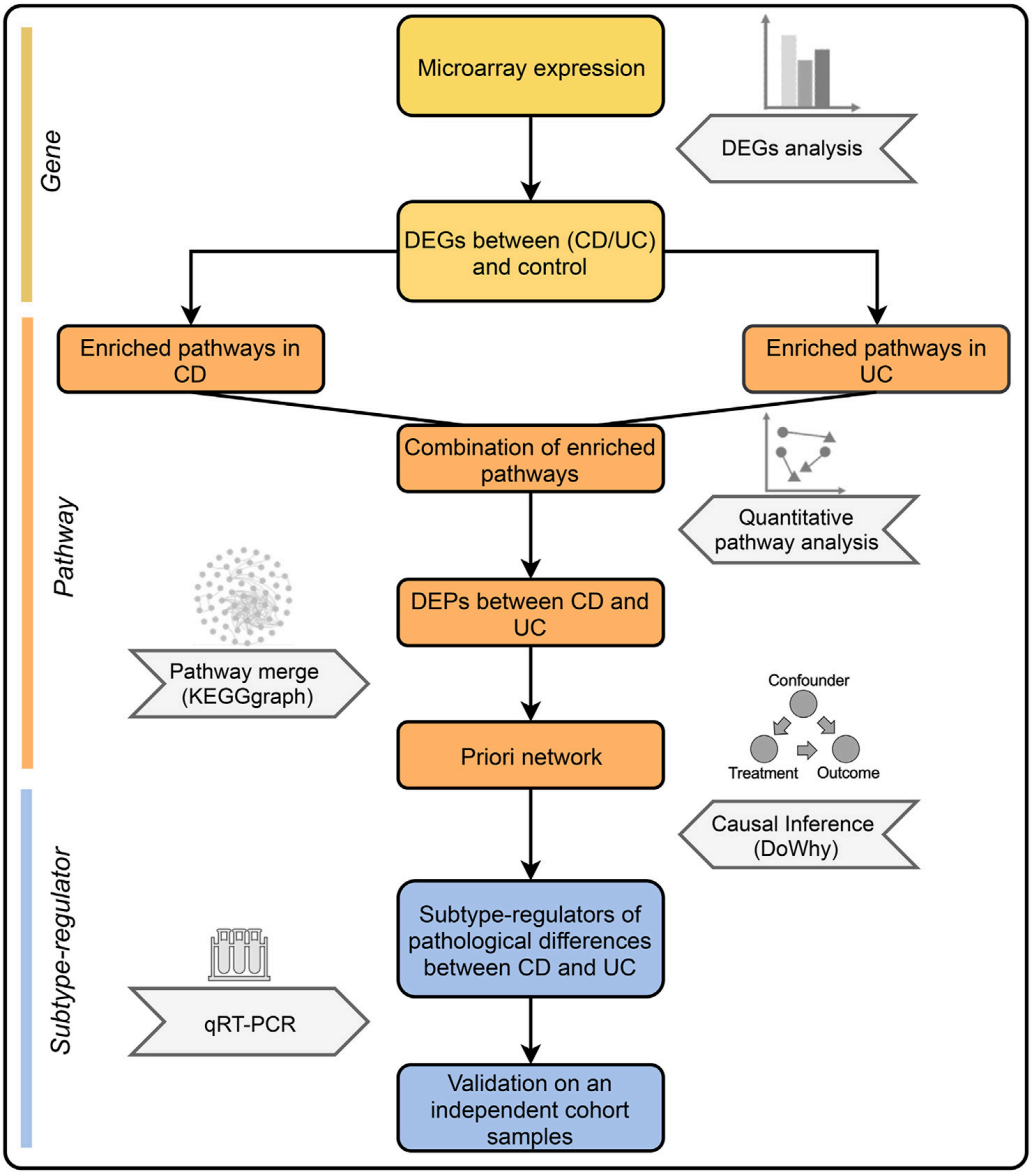
Overall workflow of this study. First, DEGs between IBD and control groups were identified. Next, the combination of enriched pathways was compared between CD and UC by QPA to determine DEPs. Subsequently, DEPs were merged into a priori network, which was used to identify the subtype-regulators using causal inference. Finally, validation was performed by qRT-PCR with an independent cohort.

Here, we took a new approach that integrates the quantitative pathway analysis (QPA) and causal inference. QPA is an approach that retains information of genes in pathway analysis and compares pathways between groups through dimension reduction and mapping of data. QPA has been successfully applied in several studies, demonstrating its advantage over the method of pathway enrichment analysis (Ren et al., 2018; Gao et al., 2020). With QPA, we identified differentially expressed pathways (DEPs) between IBD and control. Based on the priori network constructed with DEPs, causal inference was performed to identify the key factors that drive the different pathogenesis of CD and UC. Figure 1 is the overall workflow of this study.

## Materials and Methods

### Study Design

Datasets GSE38713 (Planell et al., 2013) and GSE52746 (Leal et al., 2015) generated using Affymetrix Human Genome U133 Plus 2.0 Array were selected as a discovery cohort to identify the DEGs between IBD and control groups. The selected datasets were generated in the same laboratory, minimizing potential batch effects due to different experimental conditions. A total of 10 CD, 15 UC, and 13 control samples were selected from the two datasets according to the following inclusion criteria: similar sample sizes of different groups, similar female to male ratio, and age ranges (Supplementary Tables S1, S2). Additionally, we selected transcriptome data of colonic tissues from an independent cohort (GSE111889) as a validation cohort (25 of CD, 22 of UC, and six of control).

Quantitative real-time polymerase chain reaction (qRT-PCR) was performed on samples of an independent cohort. Patients with an established diagnosis of CD and UC were included in the study. The subjects excluded from IBD based on endoscopic and histopathologic findings were classified as “non-IBD”controls. Potential participants were excluded if they were unable to or did not consent to provide tissue, were pregnant, were diagnosed with indeterminate colitis, or had an acute gastrointestinal infection. The tissue samples (*N* = 36 for CD, *N* = 13 for UC, and *N* = 33 for control, Supplementary Tables S3, S4) were collected at the time of colonoscopy. Sigmoid colon biopsies were obtained and stored in a freezer at −80°C after soaking with RNAlater (Sigma-Aldrich). The study was approved by the Institutional Review Board at The Sixth Affiliated Hospital of Sun Yat-sen University, Guangzhou, and each participant provided informed consent.

### Preprocessing of Microarray Data

MicroArray Suite 5 method was used to process the microarray raw data. “Median values of housekeeping genes adjustment” (Vandesompele et al., 2002) was used to eliminate the batch effects from two datasets. First, analysis of variance (ANOVA) was performed to identify stable reference genes among CD, UC, and control groups from the 3,804 reported housekeeping genes (Eisenberg and Levanon, 2013). Subsequently, according to the descending order of Benjamini and Hochberg adjusted *P* of ANOVA, top 100 housekeeping genes were defined as correcting reference genes (CRGs), which were used to eliminate batch effects. Afterward, a sample_(i)_ was randomly selected as a reference sample. To correct the expression of sample_(j)_, the ratio of CRG median values of sample_(i)_ and sample_(j)_ was defined as the correcting coefficient for the sample_(j)_. Finally, the expressions of genes in each sample were corrected by multiplying by the corresponding correcting coefficient, as detailed in the following formulas:

$$coefficient_{\left(j\right)}=\frac{median\left(CRGs\;expression_{\left(i\right)}\right)}{median\left(CRGs\;expression_{\left(j\right)}\right)};$$ (1)

$$corrected\;expression_{\left(j\right)}=\;expression_{\left(j\right)}\times coefficient_{\left(j\right)}\;.$$ (2)

### Differentially Expressed Gene (DEG) Analysis

Considering the non-normal distribution of the transcriptome data, non-parametric Kruskal–Wallis tests were used to test whether samples were originated from the same distribution. Subsequently, post hoc Dunnett’s tests were performed to determine DEGs between IBD (CD or UC) and control groups (|log2(Fold Change)| > 1 and *p* < 0.05).

### Quantitative Pathway Analysis (QPA)

QPA identifies DEPs among groups by projecting high-dimensional pathway data onto the same dimension for comparison. This approach has been successfully applied in previous studies (Ren et al., 2018; Gao et al., 2020). Here, QPA was used to examine the differences between CD and UC at the pathway level. Briefly, we first performed pathway enrichment via clusterProfiler (Yu et al., 2012) against the KEGG (Kyoto Encyclopedia of Genes and Genomes) database. Then, the combination of those enriched pathways between IBD (CD or UC) and control groups was compared between CD and UC. QPA was then performed as previously described (Gao et al., 2020), including 3 steps: pathway quantification, pathway comparison, and significance evaluation by the permutation test, which generates the list of DEPs between CD and UC.

### IBD Subtype-Regulator Analysis Using Causal Inference

We performed causal inference using the DoWhy (https://microsoft.github.io/dowhy/) causal inference framework to identify IBD subtype-regulators that mediate different pathogenesis of CD and UC, following an algorithm described previously (Wu et al., 2020). Based on the priori knowledge of biological interaction relationship from KEGG database, we constructed a priori network by merging all DEPs *via* KEGGgraph R package (Zhang and Wiemann, 2009). The causal effects of the DEGs between IBD (CD or UC) and control groups in the priori network were assessed with causal estimate values (CEV) of genes on driving different IBD subtypes. CEV represents the amount of change in the outcome value after intervention and the change of the treatment. Furthermore, *p* values were computed by the permutation test with 1,000 permutations to determine significant causalities. Genes with significant statistical differences between IBD subtypes and significant causal effects (*p* < 0.05) were considered as IBD subtype-regulators.

### Messenger RNA Expression by qRT-PCR

Total RNA was isolated using the Tissue RNA Purification Kit Plus (ES Science). RNA was reverse transcribed to cDNA using the Fast Reverse Transcription kit (ES Science) following the manufacturer’s instruction. qRT-PCR assays were performed using LightCycler 96 (Roche). The reaction volume was 10 μl in total including 1 μl cDNA, 0.5 μl forward and reverse primer pairs, 5 μl FastStart Essential DNA Green Master (Roche), and 3.5 μl RNase free water. The primer sequences of each gene were listed in Supplementary Table S5. β-Actin was used as the housekeeping gene. The relative expressions of these genes were calculated using the 2^−ΔΔCT^ method.

### Statistical Analysis

Differences among 3 groups were tested using one-way analysis of variance and post hoc Dunnett’s multiple comparison test. *p* value less than 0.05 was considered significant.

## Results

### Microarray Dataset Characterization

**FIGURE 2:**
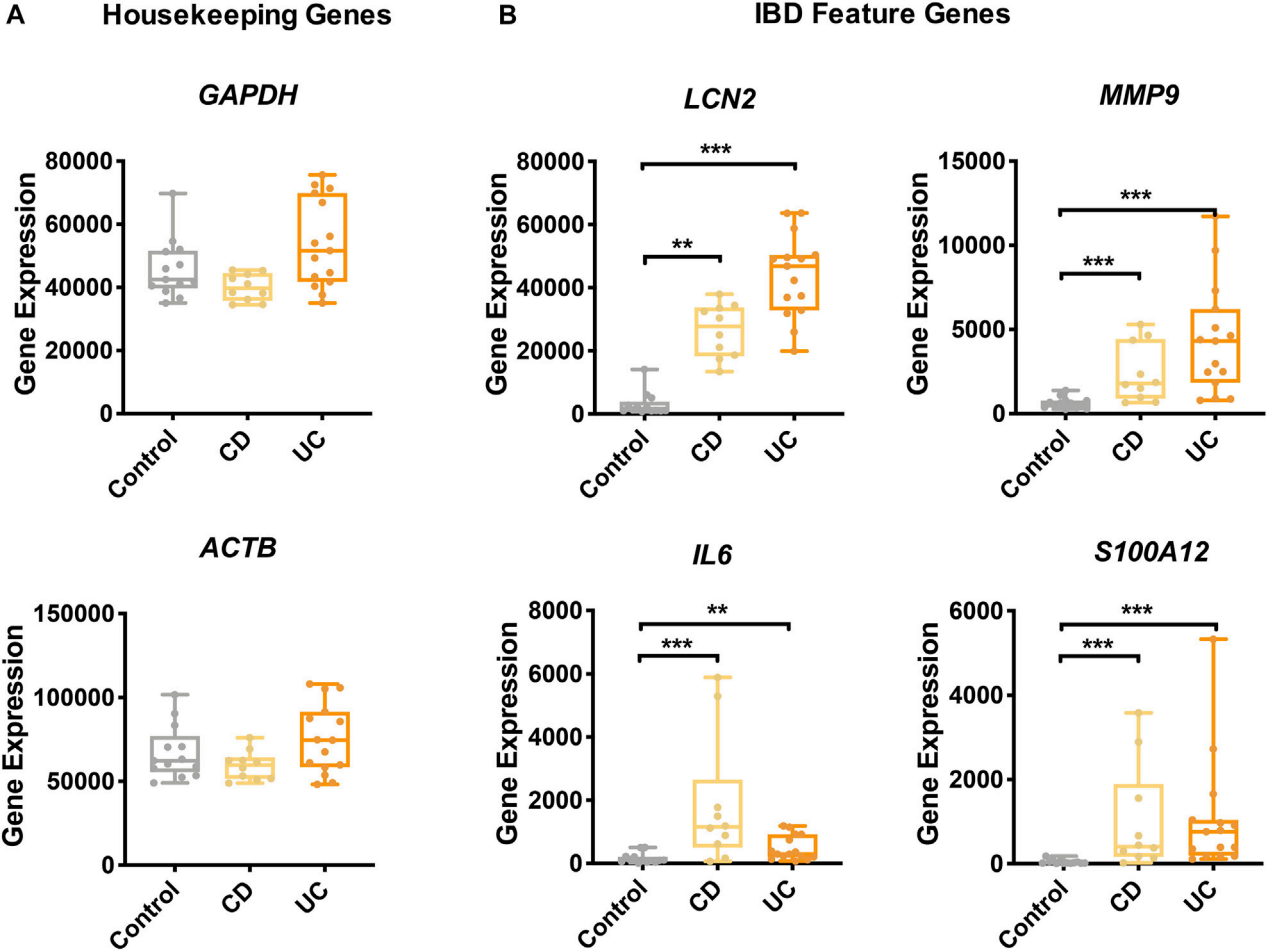
Gene expression of IBD feature genes and housekeeping genes. The expression of housekeeping genes **(A)** and feature genes of IBD **(B)** was compared among the study groups. Yellow, CD; orange, UC; gray, control. Data are presented as mean ± standard error. *: *p* < 0.05, **: *p* < 0.01, ***: *p* < 0.001, Dunnett’s test.

To examine the gene expression profiles of IBD, two microarray datasets of colonic biopsies were selected: GSE38713 and GSE52746 (Planell et al., 2013; Leal et al., 2015). To eliminate potential batch effects, we normalized gene expressions using the method of “median values of housekeeping genes adjustment” with CRGs. To validate this approach and to ascertain the quality of the data, we examined the adjusted expression levels of the housekeeping genes and some of the known IBD feature genes. As expected, the expression levels of two housekeeping genes, namely, glyceraldehyde 3-phosphate dehydrogenase (*GAPDH*) and actin beta (*ACTB*) were similar between IBD and control groups (Figure 2A), while the expression of four IBD feature genes, namely, lipocalin-2 (*LCN2*) (Cayatte et al., 2012), matrix metallopeptidase 9 (*MMP9*) (Farkas et al., 2015), interleukin 6 (*IL6*) (Atreya and Neurath, 2005), and S100 calcium-binding protein A12 (*S100A12*) (Nahidi et al., 2011) was highly elevated in CD and UC (Figure 2B).

### The DEGs Between IBD and Control Groups and the DEG-Enriched Pathways

**FIGURE 3:**
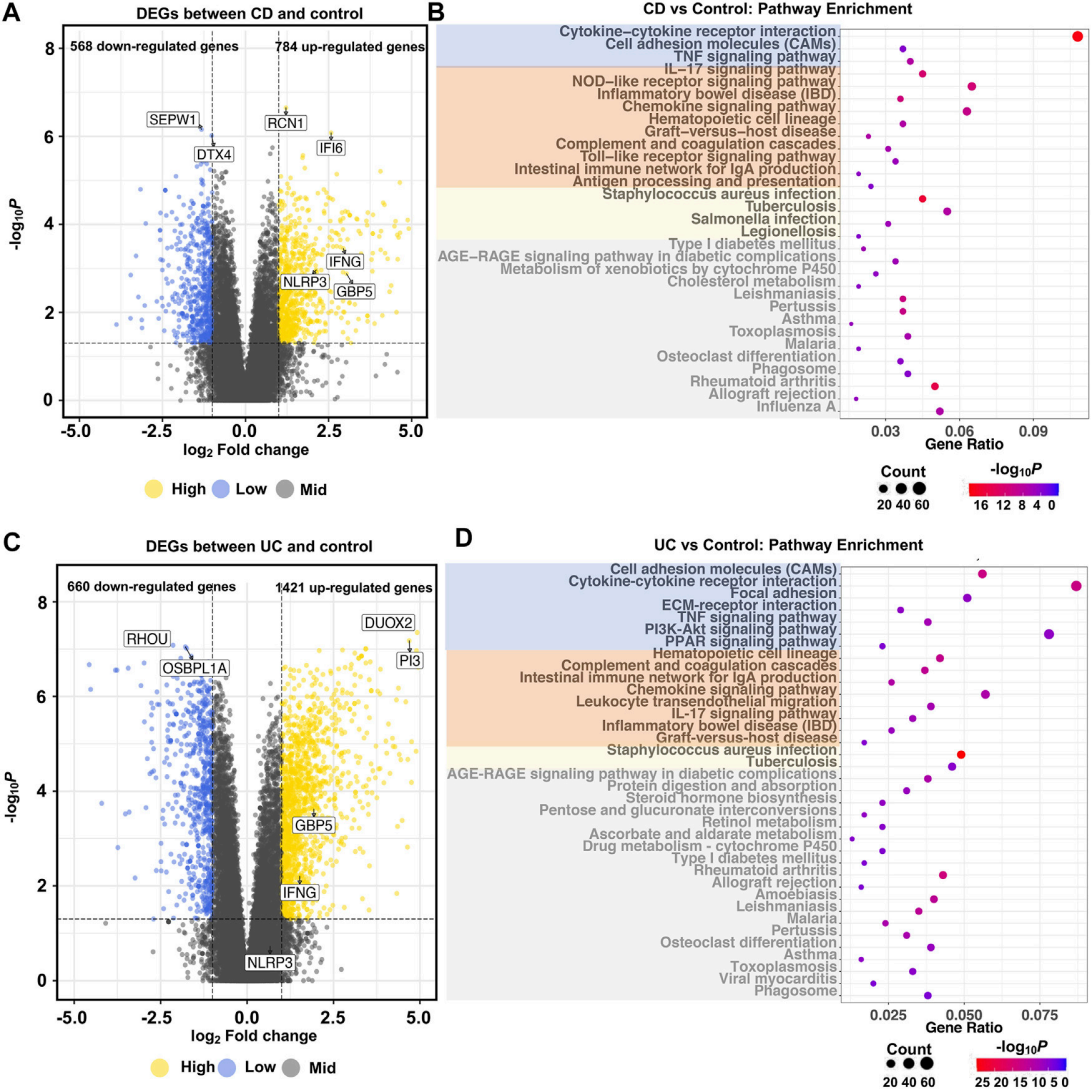
Global transcriptional changes of gene profiles and KEGG pathway enrichment in IBD. Volcano plots show the differentially expressed genes between CD **(A)**, UC **(C)**, and control groups (|log2Fold Change| >1 and adjusted *p* < 0.05). Dot plots show the pathways significantly enriched with DEGs between CD **(B)**, UC **(D)**, and control groups (adjusted *p* < 1.0e-3). *N* = 10, 15, and 13 for the CD, the UC, and the control groups, respectively. In the dot plots, signaling molecules interaction and immunity- and bacterial infection-related pathways are marked by blue, orange, and yellow, respectively. Irrelevant enriched pathways are marked by gray.

To identify the potential pathological mechanisms of IBD, we compared the transcriptome profiles of IBD (CD or UC) and control groups. At the gene level, compared to the control group, there were 1,352 and 2081 genes differentially expressed in CD and UC, respectively. In the CD group, among the 784 up-regulated genes, *RCN1* and *IFI6* were the most significantly elevated DEGs, while *SEPW1* and *DTX4* were the most significantly decreased DEGs among the 568 down-regulated genes (Figure 3A). Similarly, in the UC group, *DUOX2* and *PI3* were the most significantly elevated DEGs out of the 1,421 up-regulated genes, whereas *RHOU* and OSBPL1A were the most significantly decreased DEGs among the 660 down-regulated genes (Figure 3C). Among all the DEGs, there were a total of 657 DEGs (Supplementary Figure S1A), including 533 up-regulated (Supplementary Figure S1B) and 112 down-regulated genes (Supplementary Figure S1C), that were common in CD and UC groups. Notably, the majority of the DEGs were CD- or UC-specific, suggesting the distinct pathological mechanisms of the two IBD subtypes.

At the pathway level, pathway enrichment analysis identified 58 (Figure 3B) and 92 KEGG pathways (Figure 3D) based on the CD- and UC-specific DEGs, respectively. In contrast to the DEG results, most of the enriched pathways were common in CD (52 out of 58) and UC (52 out of 92) (Supplementary Figure S1D). These common pathways are related to signaling molecules interaction, or signaling transduction, such as cytokine–cytokine receptor interaction, cell adhesion molecules (CAMs), and bacterial infection-related pathways, such as *tuberculosis*, legionellosis, and *Staphylococcus aureus* infection. Importantly, pathways related to immune and inflammatory reactions, including the IL-17 signaling pathway, NOD-like receptor signaling pathway, toll-like receptor signaling pathway, and chemokine signaling pathway were enriched in both CD and UC. These similar biological functions reflected the common inflammatory characteristics of CD and UC. On the other hand, some inflammatory-related pathways exhibited different activities between CD and UC. For example, the NOD2 signaling pathway was only enriched in CD. This is interesting as NOD2 is an important innate immune sensor implicated in host defense in CD (Strober and Watanabe, 2011). Some pathways such as ECM-receptor interaction and PPAR signaling pathway were only enriched in UC. ECM remodeling is a decisive feature of the progression of IBD, and ECM degradation by proteases could participate in the dysfunction of intestinal barrier and inflammation in IBD (Petrey and de la Motte, 2017). PPARγ, a key molecule in the PPAR signaling pathway, is a nuclear receptor that drives bacterial-induced inflammation (Dubuquoy et al., 2006; Dou et al., 2015). These differential pathways between CD and UC suggest that IBD subtypes are different in immune and intestinal barrier-related pathomechanisms.

### DEPs Between CD and UC

**FIGURE 4:**
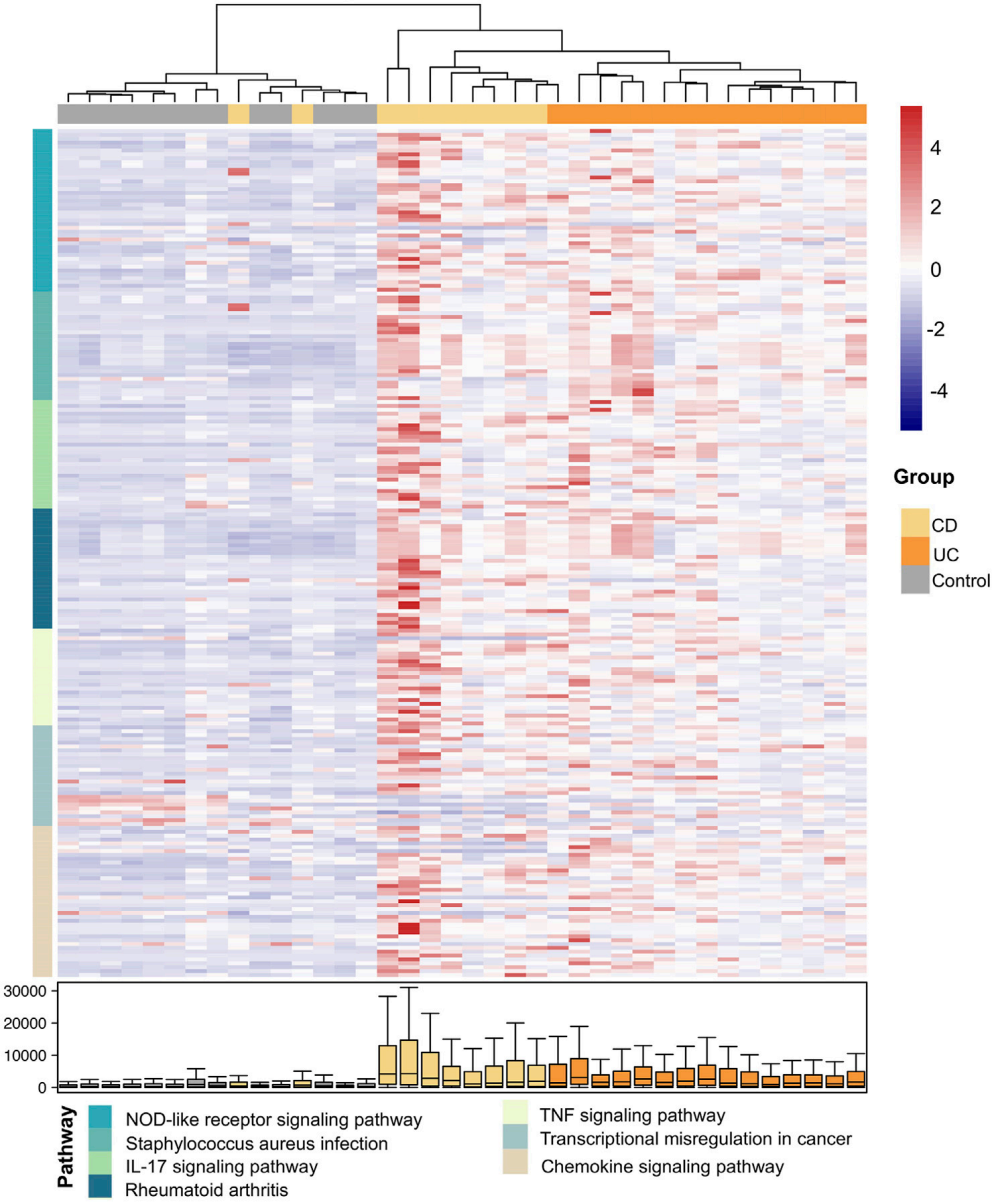
Expression heatmap of the DEGs in the DEPs between CD and UC. The samples and the DEGs are presented in columns and rows, respectively. The color of the DEGs in rows indicates different DEPs as specified in the following (pathway). The color bar on the right side indicates the scaled expressions of these genes in each sample. The distribution of the expressions for each DEG is shown by boxplot at the bottom.

Next, we investigated the differences at the pathway level between CD and UC *via* QPA. Here, we began with the pathways identified from the enrichment analysis, and a total of 98 pathways were included, which is the combination of enriched pathways in CD and UC. According to QPA, seven out of 98 pathways were significantly differentially expressed between CD and UC, including the NOD-like receptor signaling pathway, *Staphylococcus aureus* infection, IL-17 signaling pathway, rheumatoid arthritis, TNF signaling pathway, transcriptional misregulation in cancer, and chemokine signaling pathway (Supplementary Table S6). The DEPs between CD and UC were predominantly related to cytoplasmic recognition, pro-inflammatory cytokines, and cytokine interactions, which play important roles in innate immunity and inflammatory responses. We, then, examined the expression of individual DEGs in the DEPs (Figure 4). The samples were clearly clustered into three clusters consistent with the disease status, which confirmed the correlation of the DEPs with the subtypes of IBD. Compared to the control group, the expression levels of these DEGs were generally higher in IBD (CD and UC). In addition, the gene expressions of some pathways in CD were higher than those in UC, such as genes in the NOD-like receptor signaling pathway, IL-17 signaling pathway, and chemokine signaling pathway. The distinct expression levels of these DEGs in the DEPs between CD and UC suggested that these genes are related to the pathological differences between IBD subtypes.

### IBD Subtype-Regulators

**FIGURE 5:**
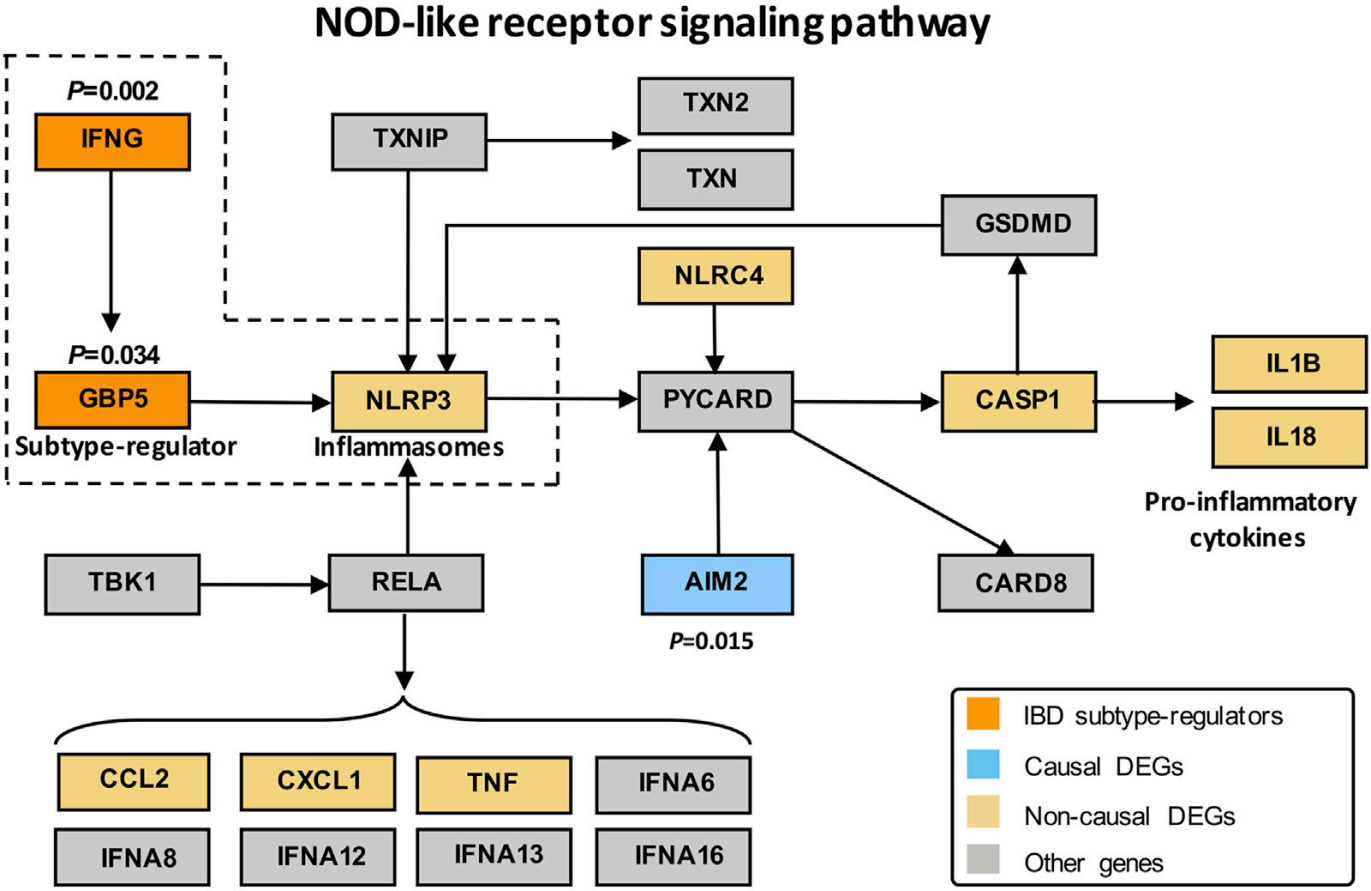
Subnetwork of the DEPs KEGG network with *IFNG*, *GBP5*, and *NLRP3*. Orange, blue, yellow, and gray represent subtype-regulators, causal DEGs, non-causal DEGs, and other genes, respectively, in DEPs. DEGs refer to differentially expressed genes between IBD and control groups. Subtype-regulators are DEGs with *p* < 0.05 in the causal effect significance test (see Method for detail) and showed statistical differences between CD and UC; causal DEGs represent DEGs with *p* < 0.05 in the causal effect significance test; non-causal DEGs represent DEGs with *p* > 0.05 in the causal effect significance test.

To identify the regulators that drive the differential pathogenesis of IBD subtypes, we performed causal inference against a priori network with established regulation relationships among genes. The priori network is composed of 709 nodes and 2,146 edges (Supplementary Figure S2) based on the DEPs generated in QPA. As a result, 27 DEGs were identified as causal DEGs (*p* < 0.05) responsible for the differences between CD and UC. The causal estimate values (CEVs) of the genes were shown in Supplementary Table S7. We, then, defined eight causal DEGs that were differentially expressed between subtypes as IBD subtype-regulators (Supplementary Table S8), including guanylate-binding protein 5 (*GBP5*), defensin beta 1 (*DEFB1*), interferon gamma (*IFNG*), C–X–C motif chemokine ligand 10 (*CXCL10*), lymphotoxin beta (LTB), prostaglandin-endoperoxide synthase 2 (*PTGS2*), suppressor of cytokine signaling 3 (*SOCS3*), and C–C motif chemokine ligand 3 (*CCL3*). Combined the results of DEP analysis with those from causal inference analysis, we found that the NOD-like receptor signaling pathway, as the most significant DEPs, was enriched with three subtype-regulators, namely, *IFNG*, *GBP5*, and *NLRP3* that work together from upstream to downstream in the regulation of immune response, which indicated the important roles of the three subtype-regulators in the pathway. Therefore, we extracted and depicted a subnetwork of the priori DEP network by retrieving *IFNG*, *GBP5*, and *NLRP3* and their two most adjacent genes in the NOD-like receptor signaling pathway (Figure 5).

**FIGURE 6:**
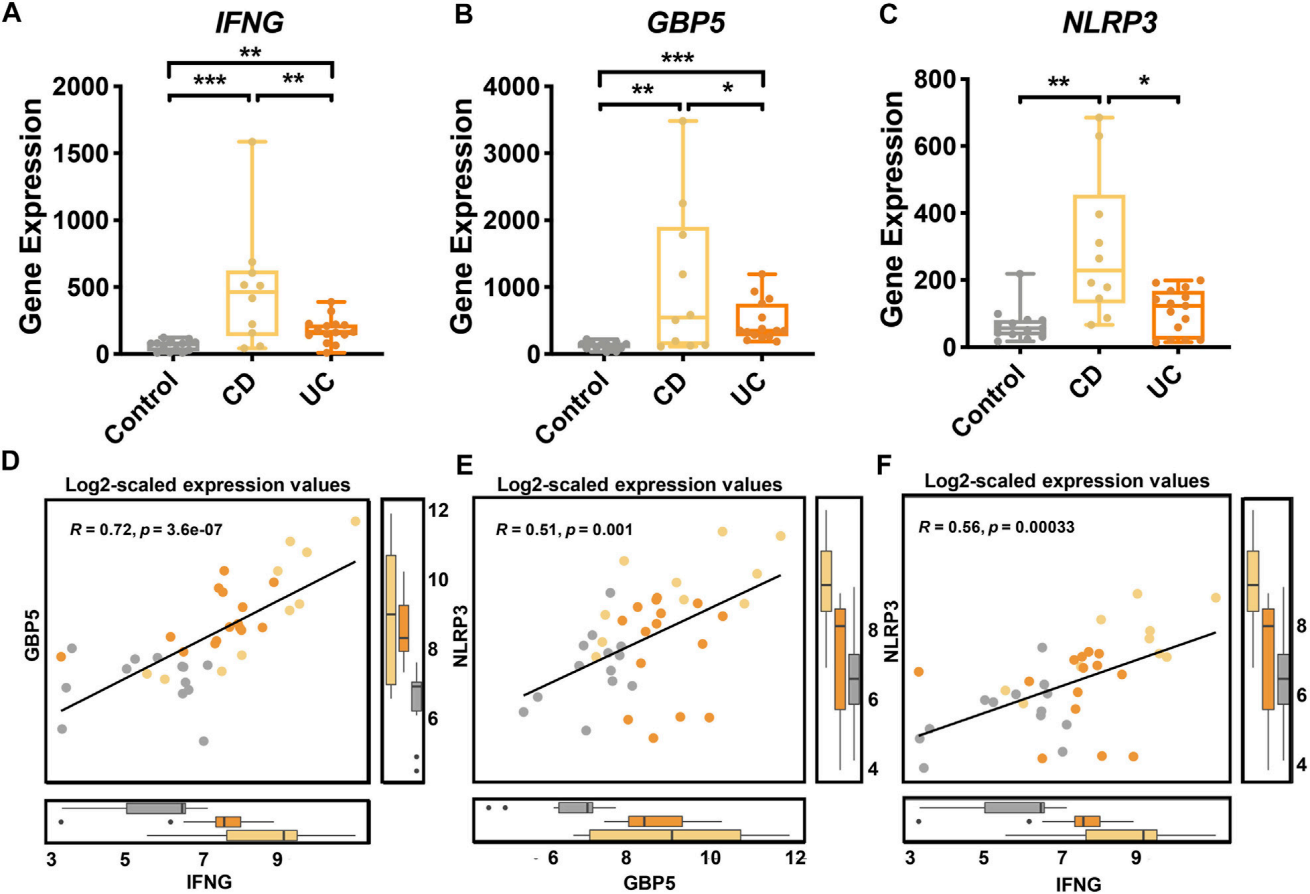
Gene expression of *IFNG*, *GBP5*, and *NLRP3* in healthy controls, CD, and UC patients according to transcriptome analysis. The expression of **(A)** *IFNG*, **(B)** *GBP5*, and **(C)** *NLRP3* genes in the control, CD, and UC groups. Yellow, CD; orange, UC; gray, control. All data are presented as mean ± standard error. *: *p* < 0.05; **: *p* < 0.01; ***: *p* < 0.001. *N* = 10, 15, and 13 for the CD, the UC, and the control groups, respectively. **(D)** Linear regression between *IFNG* and *GBP5* based on log2-scaled expression values (Spearman; R = 0.72, *p* = 3.6e-07). **(E)** Linear regression between *GBP5* and *NLRP3* based on log2-scaled expression values (Spearman; R = 0.51, *p* = 0.001). **(F)** Linear regression between *IFNG* and *NLRP3* based on log2-scaled expression values (Spearman; R = 0.56, *p* = 0.00033).

For a closer examination of the expression of *IFNG*, *GBP5*, and *NLRP3*, our transcriptome data showed that *IFNG* (Figure 6A), *GBP5* (Figure 6B), and *NLRP3* (Figure 6C) were significantly up-regulated in CD than those in UC and control groups. Additionally, the expressions of *GBP5* were positively correlated with those of *IFNG* (Figure 6D) and *NLRP3* (Figure 6E), respectively (Spearman, *p* < 0.05). The expressions of *IFNG* and those of *NLRP3* were also positively correlated (Figure 6F). Regression analysis was also performed with *IFNG, GBP5*, and *NLRP3* in CD, UC, and controls, respectively (Supplementary Figure S3). Thus, our data indicated a sequential cause of relationships from *IFNG* to *GBP5,* and then to *NLRP3*. With the validation dataset, the expression of *IFNG, GBP5*, and *NLRP3* was consistent with that in the discovery datasets (Supplementary Table S9), and the regression analysis also demonstrated similar relationships among these three genes (Supplementary Figure S4).

Furthermore, we examined the expression of pro-inflammatory cytokines interleukin 1 beta (*IL1B*) and interleukin 18 (*IL18*) that are at the endpoint of NLRP3 inflammasome activation. According to our transcriptome data, the expression of both *IL1B* and *IL18* was significantly higher in CD than those in UC and control groups, and both *IL1B* and *IL18* were positively correlated with *NLRP3* (Supplementary Figure S5). These results support a unique role for NLRP3 inflammasome in CD compared to UC.

### Validation of the Differential Expression of IBD Subtype-Regulators by qRT-PCR

**FIGURE 7:**
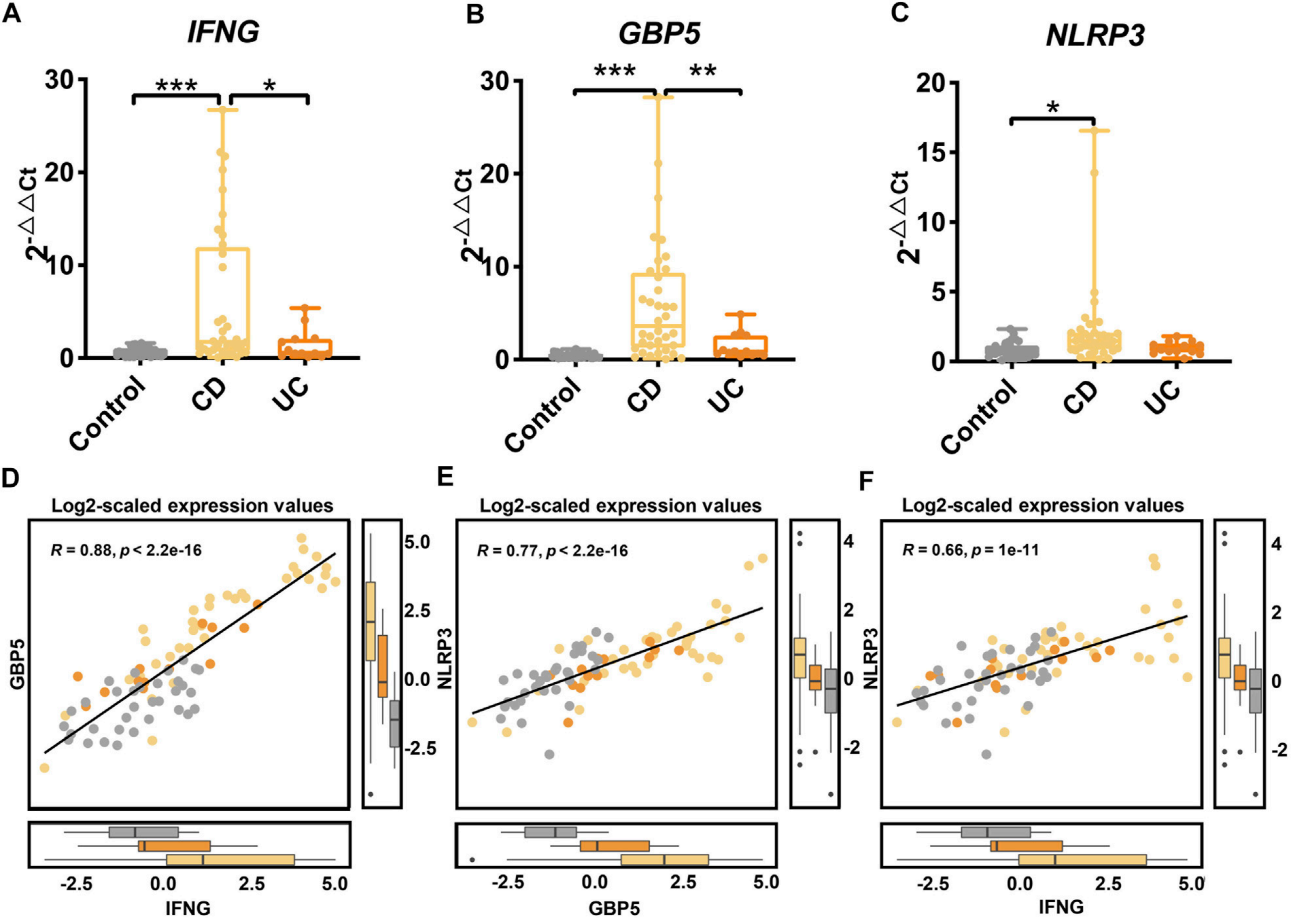
Gene expression of *IFNG*, *GBP5*, and *NLRP3* in an independent cohort of healthy controls, CD, and UC patients as determined by qRT-PCR. The gene expressions of **(A)** *IFNG*, **(B)** *GBP5*, and **(C)** *NLRP3* in the control, the CD, and the UC patients. Data are presented as mean ± standard error. *p < 0.05, **p < 0.01, and ***p < 0.001. *N* = 36, 13, and 33 for the CD, the UC, and the control groups, respectively. **(D)** Linear regression between *IFNG* and *GBP5* based on log2-scaled expression values (Spearman, R = 0.88, *p* < 2.2e-16). **(E)** Linear regression between *GBP5* and *NLRP3* based on log2-scaled expression values (Spearman, R = 0.77, *p* < 2.2e-16). **(F)** Linear regression between *IFNG* and *NLRP3* based on log2-scaled expression values (Spearman, R = 0.66, *P* = 1e-11).

We validated the differential expression of *IFNG*, *GBP5*, and *NLRP3* using qRT-PCR on an independent cohort of healthy controls, CD and UC patients (Supplementary Table S9). Compared to the UC and the control groups, the expression of the *IFNG* was significantly higher in the CD group (Figure 7A; *p* < 0.05 and *p* < 0.001, respectively). The expression of *GBP5* showed a similar pattern in three groups, and was significantly higher in the CD group than that in the UC and the control group (Figure 7B; *p* < 0.01 and *p* < 0.001, respectively). The expression of *NLRP3* was significantly higher in the CD group than that in the control group (Figure 7C; *p* < 0.05). Overall, the PCR results were consistent with the transcriptome data that the up-regulation of *IFNG*, *GBP5*, and *NLRP3* was more significant in CD than that in UC. Again, significant correlations in gene expression levels were observed among *IFNG*, *GBP5*, and *NLRP3* (Figures 7D–F). With qRT-PCR data, regression analysis showed that correlations among *IFNG, GBP5*, and *NLRP3* in CD were more significant than those in UC (Supplementary Figure S6).

## Discussion

In the present study, we identified *IFNG* and *GBP5* as IBD subtype-regulators that play key roles at the early steps of the pathogenesis of CD and UC using causal inference. *IFNG* and *GBP5* may drive the pathological differences between CD and UC by up-regulating *NLRP3.* That is, *NLRP3* is more intensely induced by *IFNG/GBP5* in CD than that in UC. These findings facilitate our understanding of the pathological mechanisms of CD and UC and provide a foundation for the personalized medicine for IBD patients. We identified *IFNG* and *GBP5* as IBD subtype-regulators.

Many studies have investigated possible pathological mechanisms of CD and UC (Ramos and Papadakis, 2019; Kaur and Goggolidou, 2020). However, it is unclear what triggers the phenotypic differences between CD and UC. In transcriptome studies, the biological relationships could be masked by irrelevant impacts (confounders) and indirect effects (mediators) (Vansteelandt and Vanderweele, 2012). Thus, it is difficult to identify IBD subtype-regulators through simple statistical analysis such as correlation analysis. Causal inference is a technique used for extracting causal estimates from observational data with strong exactness and interpretability, providing key insights by studying how behaviors or treatments affect the outcomes of interest, and has been increasingly employed to investigate the relationship between microbiota and host diseases (Goren et al., 2021; Lv et al., 2021). To better explore the complex mechanisms within IBD, we applied causal inference to analyze transcriptome data and unveil the potential regulators of IBD subtypes in this study.

In order to obtain disease-related priori knowledge for causal inference, we used QPA to identify latent differences between CD and UC at the pathway level. The NOD-like receptor signaling pathway, IL-17 signaling pathway, TNF signaling pathway, and chemokine signaling pathway were demonstrated as DEPs between CD and UC. These results indicate that differences between CD and UC indeed exist and they may contribute to the different clinical presentations between CD and UC. Among the DEPs, the most significant DEP, the NOD-like receptor signaling pathway (*p* = 0.020) plays a crucial role in maintaining the innate immune response to microbiota in the intestine by inducing inflammatory and chemotactic molecules such as *IL1B* and *IL18* (Franchi et al., 2009). Many studies have demonstrated that NOD-like receptors (NLRs) are closely related to the the intestinal mucosal barrier which prevents the invasion of gut microbiota (Zhong et al., 2013). Particularly, *NLRP3,* a member of NLRs family, has received much attention in the studies of the pathological mechanism of IBD (Wang et al., 2020). *NLRP3* is a critical gene in the NOD-like receptor signaling pathway that contributes to the formation of the *NLRP3* inflammasome in immune cells, which could cause *CASP1*-mediated proteolytic activation of IL1B and IL18, and induces inflammation, and pyroptotic cell death in IBD (Tourkochristou et al., 2019). Our transcriptome data also indicated that both *IL1B* and *IL18* were highly expressed in CD. In contrast, *IL18*, but not *IL18* was highly expressed in UC. The positive correlations of the *NLRP3* gene with its downstream pro-inflammatory cytokines (IL1B and IL18) provide further evidence for the up-regulation of *NLRP3* inflammasome activity in CD. Thus, these results demonstrated that the NLRP3 inflammasome was more activated in CD than that in UC. In line with our observations, a previous study reported the higher activation of *NLRP3* inflammasome in CD patients than in UC and control (Lazaridis et al., 2017). These observations support a role for *NLRP3* in distinct inflammatory activities between CD and UC. Moreover, many studies have revealed that microbiome composition could be impacted by *NLRP3* inflammasome (Yao et al., 2017; Zhang et al., 2019), which demonstrated its important role in regulating microbiome balance in the intestine.

However, *NLRP3* was not identified as an IBD subtype-regulator with a CEV = 4.78e-6 and a *p* = 0.45 after eliminating the irrelevant impacts and indirect effects from other genes in the regulation network. Instead, the upstream genes *IFNG* (CEV = 0.004, *p* = 0.002) and *GBP5* (CEV = 0.0009, *p* = 0.034) showed strong causal effects on IBD subtypes. Our result is supported by the reports that *IFNG* induces *GBP5* (Fujiwara et al., 2016), which in turn activates the *NLRP3* inflammasome with key roles in innate immunity and overall inflammation (Bergantz et al., 2019).

*IFNG* is a signature pro-inflammatory cytokine secreted by infected cells. It activates the innate immune response that promotes not only cytokine production but also natural killer cell functions and antigen presentation (Kopitar-Jerala, 2017). The previous study has demonstrated that *IFNG* plays key roles in the initiation of IBD, and the *IFNG* deficient mice did not develop DSS-induced colitis (Ito et al., 2006). Importantly, the increased levels of *IFNG* were found in the inflamed mucosa of CD patients but a normal level of *IFNG* was found in UC (Neurath et al., 2002). On the other hand, *GBP5* is highly inducible by pro-inflammatory cytokines (Fujiwara et al., 2016), including *IFNG*, and is a marker of *IFNG*-induced activated macrophages. Our results showed that *IFNG* and its downstream gene *GBP5* were highly expressed in CD than in UC and control. Therefore, the different expression levels of *IFNG* and *GBP*5 between CD and UC could be the key factor driving different disease manifestations of CD and UC. Moreover, many studies have revealed that the microbiome composition was different between CD and UC (Sankarasubramanian et al., 2020), which might be the consequence of distinct host immunity. When recognizing microbial antigens, different intensities of host immune responses might lead to the differences of immune cells activation, cytokines secretion, and severity of inflammation, which further impact the environment of gut microorganisms. Therefore, the differences in immune responses caused by *IFNG, GBP5*, and *NLRP3* may contribute to the differences in microbiome composition between IBD subtypes.

Interestingly, *DEFB1*, *CCL3*, *CXCL10*, *LTB*, *PTGS2*, and *SOCS3* were also identified as IBD subtype-regulators. In our transcriptome data, the expression of *CCL3*, *CXCL10*, *LTB*, *PTGS2*, and *SOCS3* were up-regulated, whereas the expression of *DEFB1* was down-regulated in CD and UC compared to the control. *DEFB1* is known as a multifunctional mediator in infection and inflammation. The expression of *DEFB1* can be up-regulated by LPS, IL-1β, IFN-γ, and arginine. However, a study has demonstrated that the impaired *DEFB1* expression in colonic CD is not linked to inflammation-associated tissue damage (Peyrin-Biroulet et al., 2010). *CCL3* is a chemokine related to neutrophils. The early release of *CCL3* by neutrophils plays roles in the development of a protective immune response (Charmoy et al., 2010). Similarly, *CXCL10* is a CXC chemokine in the DEP chemokine signaling pathway that is highly expressed in both CD and UC (Banks et al., 2003). CXCL10 contributes to systemic inflammation, colonic Th1 recruitment, and adaptive immunity in colitis. Our transcriptome data showed that the expression levels of both *CCL3* and *CXCL10* were increased significantly in CD compared to UC, suggesting that the differential expressions of these genes have strong causal relationships with different innate immune responses between CD and UC. *LTB* is a pro-inflammatory lipid mediator that could recruit leukocytes to the injury site and plays a pathogenic role in inflammation. *PTGS2*, also known as COX2, plays a critical role in regulating inflammatory responses (Hellmann et al., 2015). However, the roles of *LTB* and *PTSG2* in IBD still remain undefined. Moreover, we found that *SOCS3* increased in both CD and UC, although the over-expression of *SOCS3* has been reported to be important for limiting inflammatory responses (Li et al., 2012). The increased expression of *SOCS3* might be a response to the excessive inflammation in the intestine instead of a cause for different pathogenesis of CD and UC. In summary, these IBD subtype-regulators provide new insight into the diagnosis of IBD subtypes and facilitate our understanding of the pathological mechanisms of CD and UC.

We acknowledge the limitation that the sample size of this study is relatively small due to the lack of data with sufficient quality. Although it would not affect our conclusions, further evidence from larger IBD cohorts is needed. Nevertheless, with the application of QPA and causal inference, this study provides a paradigm of identifying key regulators of disease based on a quantitative causal analysis of transcriptome data, enabling and promoting future work that aims to uncover causal mechanisms of disease.

## Conclusion

Our study suggests that *IFNG* and *GBP5* were IBD subtype-regulators and trigger more intense innate immunity and inflammatory responses in CD than those in UC. Our findings reveal pathomechanical differences between CD and UC that may contribute to personalized treatment for CD and UC. Additionally, the protocol of causal inference analysis proposed in this study could be generalized for other transcriptome studies.

## Data Availability

The datasets presented in this study can be found in online repositories. The names of the repository/repositories and accession number(s) can be found in the article/Supplementary Material.

## Supplementary Material

Supplementary Figure S1 DEGs and pathways with altered activities in IBD. **(A)** All DEGs in IBD; **(B)** up-regulated DEGs in IBD; **(C)** down-regulated DEGs in IBD; and **(D)** enriched pathways in IBD.

Supplementary Figure S2 Network of DEPs KEGG. Blue, yellow, and gray represent causal DEGs, non-causal DEGs, and other genes, respectively, in DEPs. The node size represents the significance of causal effects.

Supplementary Figure S3 Regression analysis of *IFNG*, *GBP5*, and *NLRP3* in healthy controls, CD, and UC patients, respectively, according to transcriptome analysis. *N* = 10, 15, and 13 for CD, UC, and control groups, respectively. **(A–C)** Linear regression between *IFNG* and *GBP5* based on log2-scaled expression values in CD (Spearman; R = 0.89, *p* = 0.00066), UC (Spearman; R = 0.74, *p* = 0.0039), and control (Spearman; R = −0.022, *p* = 0.94). **(D–F)** Linear regression between *GBP5* and *NLRP3* based on log2-scaled expression values in CD (Spearman; R = 0.69, *p* = 0.026), UC (Spearman; R = −0.019, *p* = 0.95), and control (Spearman; R = 0.46, *p* = 0.12). **(G–J)** Linear regression between *IFNG* and *NLRP3* based on log2-scaled expression values in CD (Spearman; R = 0.76, *p* = 0.011), UC (Spearman; R = −0.19, *p* = 0.51), and control (Spearman; R = 0.45, *p* = 0.12).

Supplementary Figure S4 Gene expression of *IFNG*, *GBP5*, and *NLRP3* in healthy controls, CD, and UC patients in an independent cohort. The expression of **(A)** *IFNG*, **(B)** *GBP5*, and **(C)** *NLRP3* genes in the control, CD, and UC groups. Yellow, CD; orange, UC; gray, control. All data are presented as mean ± standard error. *: *p* < 0.05;**: *p* < 0.01;***: *p* < 0.001. *N* = 10, 15, and 13 for CD, UC, and control groups, respectively. **(D)** Linear regression between *IFNG* and *GBP5* based on log2-scaled expression values (Spearman; R = 0.9, *p* < 2.2e-16). **(E)** Linear regression between *GBP5* and *NLRP3* based on log2-scaled expression values (Spearman; R = 0.85, *p* = 4.5e-16). (F) Linear regression between *IFNG* and *NLRP3* based on log2-scaled expression values (Spearman; R = 0.81, *p* = 4.9e-13).

Supplementary Figure S5 Gene expression of *IL1B* and *IL18* in healthy controls, CD, and UC patients according to transcriptome analysis. The expression of **(A)** *IL1B* and **(B)** *IL18* genes in the control, CD, and UC groups. Yellow, CD; orange, UC; gray, control. All data are presented as mean ± standard error. *: *p* < 0.05; **: *p* < 0.01; ***: *p* < 0.001. *N* = 10, 15, and 13 for CD, UC, and control groups, respectively. **(C)** Linear regression between *NLRP3* and *IL1B* based on log2-scaled expression values (Spearman; R = 0.7, *p* = 7.8e-07). **(D)** Linear regression between *NLRP3* and *IL18* based on log2-scaled expression values (Spearman; R = 0.4, *p* = 0.014).

Supplementary Figure S6 Regression analysis of *IFNG, GBP5*, and *NLRP3* in healthy controls, CD, and UC patients, respectively, according to qRT-PCR data. *N* = 36, 13, and 33 for CD, UC, and control groups, respectively. **(A–C)** Linear regression between *IFNG* and *GBP5* based on log2-scaled expression values in CD (Spearman; R = 0.9, *p* = 1.1e-13), UC (Spearman; R = 0.88, *p* = 6.6e-05), and control (Spearman; R = 0.63, *p* = 8.6e-05). **(D–F)** Linear regression between *GBP5* and *NLRP3* based on log2-scaled expression values in CD (Spearman; R = 0.78, *p* = 2e-08), UC (Spearman; R = 0.74, *p* = 0.0039), and control (Spearman; R = 0.69, *p* = 7.6e-06). **(G–J)** Linear regression between *IFNG* and *NLRP3* based on log2-scaled expression values in CD (Spearman; R = 0.61, *p* =7.2e-05), UC (Spearman; R = 0.67, *p* = 0.013), and control (Spearman; R = 0.48, *p* = 0.0048).

## Abbreviations

ACTB, actin beta; CASP1, caspase 1; CCL3, C–C motif chemokine ligand 3; CD, Crohn’s disease; CEV, causal estimate values; CRG, correcting reference genes; CXCL10, C–X–C motif chemokine ligand 10; DEFB1, defensin beta 1; DEGs, differentially expressed genes; DEPs, differentially expressed pathways; GAPDH, glyceraldehyde 3-phosphate dehydrogenase; GBP5, guanylate-binding protein 5; IBD, inflammatory bowel disease; IFNG, interferon gamma; IL-17, interleukin 17; IL1B, interleukin 1 beta; IL18, interleukin 18; IL6, interleukin 6; LCN2, lipocalin-2; LTB, lymphotoxin beta; MMP9, matrix metallopeptidase 9; NLRP3, NLR family pyrin domain-containing 3; PTGS2, prostaglandin-endoperoxide synthase 2; PYCARD, PYD and CARD domain containing; qRT-PCR, quantitative real-time polymerase chain reaction; S100A12, S100 calcium binding protein A12; SOCS3, suppressor of cytokine signaling 3; UC, ulcerative colitis.
