# Supplementary material for: IBD Subtype-Regulators IFNG and GBP5 Identified by Causal Inference Drive More Intense Innate Immunity and Inflammatory Responses in CD Than Those in UC
Source: Front Pharmacol. 2022 Apr 6;13:869200. doi: 10.3389/fphar.2022.869200 (PMC9020454; doi:10.3389/fphar.2022.869200)
Supplement: Supplementary file 2 [file Table1.DOCX]

**Supplementary Table 1. Sample information of microarray datasets**

| **Factor** | **Crohn's disease (CD)** | **Ulcerative colitis (UC)** | **Control** |
| --- | --- | --- | --- |
| Sample size | 10 | 15 | 13 |
| Gender（Male/Female） | 7/3 | 4/11 | 8/5 |
| Age（Year） | 40.5±15.5 | 42.4±9.1 | 41.6±11.8 |
| Disease course（Year） | 6.0±5.4 | 6.6±5.2 | - |
