# Supplementary material for: IBD Subtype-Regulators IFNG and GBP5 Identified by Causal Inference Drive More Intense Innate Immunity and Inflammatory Responses in CD Than Those in UC
Source: Front Pharmacol. 2022 Apr 6;13:869200. doi: 10.3389/fphar.2022.869200 (PMC9020454; doi:10.3389/fphar.2022.869200)
Supplement: Supplementary file 3 [file Table8.DOCX]

**Supplementary Table 8. IBD subtype-regulators of the pathological differences between CD and UC**

| **Gene Symbol** | **Entrez ID** | **CD vs Control** | ***P*-value** | **UC vs Control** | ***P*-value** | **CD vs UC** | ***P*-value** |
| --- | --- | --- | --- | --- | --- | --- | --- |
|  |  | **Foldchange** |  | **Foldchange** |  | **Foldchange** |  |
|  |  |  |  |  |  |  |  |
| GBP5 | 115362 | 8.2101 ↑ | 0.0013 ** | 3.8139 ↑ | 0.0002 *** | 2.1527 ↑ | 0.0315 * |
| DEFB1 | 1672 | 0.5153 ↓ | 0.0949 | 0.1476 ↓ | 0.0000 *** | 3.4917 ↑ | 0.0137 * |
| IFNG | 3458 | 7.6943 ↑ | 0.0004 *** | 2.8725 ↑ | 0.0062 ** | 2.6786 ↑ | 0.0013 ** |
| CXCL10 | 3627 | 16.8728 ↑ | 0.0001 *** | 6.7507 ↑ | 0.0004 *** | 2.4994 ↑ | 0.0213 * |
| LTB | 4050 | 2.6664 ↑ | 0.0032 ** | 1.2567 ↑ | 0.2097 | 2.1217 ↑ | 0.0405 * |
| PTGS2 | 5743 | 17.6607 ↑ | 0.0000 *** | 4.2036 ↑ | 0.0029 ** | 4.2014 ↑ | 0.0023 ** |
| SOCS3 | 9021 | 6.6900 ↑ | 0.0003 *** | 2.5505 ↑ | 0.0154 * | 2.6230 ↑ | 0.0443 * |
| CCL3 | 6348 | 8.8005 ↑ | 0.0001 *** | 2.3032 ↑ | 0.0090 ** | 3.8210 ↑ | 0.0421 * |
| The arrows mean the up or down regulation of gene expressions. * indicates that the gene is significant differentially expressed (\|log2(FC)\|>log2(2); FDR < 0.05). FC represents fold change. *P*-value was sorted by ascending. | | | | | | | |
|  | | | | | | | |
|  | | | | | | | |
