# Supplementary material for: IBD Subtype-Regulators IFNG and GBP5 Identified by Causal Inference Drive More Intense Innate Immunity and Inflammatory Responses in CD Than Those in UC
Source: Front Pharmacol. 2022 Apr 6;13:869200. doi: 10.3389/fphar.2022.869200 (PMC9020454; doi:10.3389/fphar.2022.869200)
Supplement: Supplementary file 6 [file Table7.DOCX]

**Supplementary Table 7. Causal DEGs revealed by causal inference analysis**

| **ENTREZ ID** | **SYMBOL** | **Causal Estimate Value** | ***P*-value** |
| --- | --- | --- | --- |
| 2209 | FCGR1A | 0.0245 | 0.001 |
| 2215 | FCGR3B | 0.003 | 0.001 |
| 4616 | GADD45B | 0.0009 | 0.001 |
| 414062 | CCL3L3 | 0.0006 | 0.002 |
| 5743 | PTGS2 | 0.0007 | 0.002 |
| 3458 | IFNG | 0.004 | 0.002 |
| 9021 | SOCS3 | 0.0002 | 0.002 |
| 1906 | EDN1 | 0.0012 | 0.002 |
| 1672 | DEFB1 | 0.0027 | 0.002 |
| 3248 | HPGD | 0.0001 | 0.002 |
| 6348 | CCL3 | 0.0006 | 0.004 |
| 5468 | PPARG | 0.0007 | 0.005 |
| 4050 | LTB | 0.001 | 0.005 |
| 6349 | CCL3L1 | 0.0006 | 0.006 |
| 597 | BCL2A1 | 0.0002 | 0.006 |
| 1326 | MAP3K8 | 0.0018 | 0.007 |
| 10135 | NAMPT | 0.0001 | 0.01 |
| 3627 | CXCL10 | 0.0002 | 0.013 |
| 9447 | AIM2 | 0.0038 | 0.015 |
| 3383 | ICAM1 | 0.0023 | 0.015 |
| 6373 | CXCL11 | 0.0003 | 0.016 |
| 1493 | CTLA4 | 0.0113 | 0.016 |
| 7128 | TNFAIP3 | 0.0002 | 0.021 |
| 2633 | GBP1 | 0.0001 | 0.023 |
| 6351 | CCL4 | 0.0003 | 0.027 |
| 115362 | GBP5 | 0.0009 | 0.034 |
| 7102 | TSPAN7 | 0.0004 | 0.05 |

The causal estimate values of causal DEGs were calculated by DoWhy framework. **P-*value was sorted by ascending.
