# Supplementary material for: IBD Subtype-Regulators IFNG and GBP5 Identified by Causal Inference Drive More Intense Innate Immunity and Inflammatory Responses in CD Than Those in UC
Source: Front Pharmacol. 2022 Apr 6;13:869200. doi: 10.3389/fphar.2022.869200 (PMC9020454; doi:10.3389/fphar.2022.869200)
Supplement: Supplementary file 8 [file Table9.DOCX]

**Supplementary Table 9. Expression of *IFNG*, *GBP5* and *NLRP3* in transcriptome data and qRT-PCR validation**

| **Gene Symbol** | **Entrez ID** |  | **CD vs Control** | ***P*-value** | **UC vs Control** | ***P*-value** | **CD vs UC** | ***P*-value** |
| --- | --- | --- | --- | --- | --- | --- | --- | --- |
|  |  | **mRNA/Protein** | **Foldchange** |  | **Foldchange** |  | **Foldchange** |  |
|  |  |  |  |  |  |  |  |  |
| *IFNG* | 3458 | mRNA (discovery) | 7.6943 ↑ | 0.0004 *** | 2.8725 ↑ | 0.0062 ** | 2.6786 ↑ | 0.0013 ** |
| *IFNG* | 3458 | mRNA (validate) | 30.7315 ↑ | 0.0407 * | 4.6399 ↑ | 0.6049 | 6.6231 ↑ | 0.0166 * |
| *IFNG* | 3458 | Protein (PCR) | 9.6364 ↑ | 0.0001 *** | 2.2635 ↑ | 0.8861 | 4.2572 ↑ | 0.0189 * |
| *GBP5* | 115362 | mRNA (discovery) | 8.2101 ↑ | 0.0013 ** | 3.8139 ↑ | 0.0002 *** | 2.1527 ↑ | 0.0315 * |
| *GBP5* | 115362 | mRNA (validate) | 9.1559 ↑ | 0.0425 * | 1.9832 ↑ | 0.85666 | 4.6166 ↑ | 0.0029 ** |
| *GBP5* | 115362 | Protein (PCR) | 20.9170↑ | <0.0001 *** | 4.0018↑ | 0.7375 | 5.2268↑ | 0.0052 ** |
| *NLRP3* | 114548 | mRNA (discovery) | 4.3757↑ | 0.0011 *** | 1.5864↑ | 0.1880 | 2.7583↑ | 0.0212 * |
| *NLRP3* | 114548 | mRNA (validate) | 2.7519 ↑ | 0.0198 * | 1.6082 ↑ | 0.8831 | 1.7111 ↑ | 0.0349 * |
| *NLRP3* | 114548 | Protein (PCR) | 2.8261 ↑ | 0.0169 * | 1.1633 ↑ | 0.9813 | 2.4293 ↑ | 0.1436 |

The arrows mean the up or down regulation of gene expressions. * indicates that the gene is significant differentially expressed (|log2(FC)|>log2(2); FDR < 0.05). FC represents fold change. *P*-value was sorted by ascending.
