# Supplementary material for: IBD Subtype-Regulators IFNG and GBP5 Identified by Causal Inference Drive More Intense Innate Immunity and Inflammatory Responses in CD Than Those in UC
Source: Front Pharmacol. 2022 Apr 6;13:869200. doi: 10.3389/fphar.2022.869200 (PMC9020454; doi:10.3389/fphar.2022.869200)
Supplement: Supplementary file 9 [file Table2.DOCX]

**Supplementary Table 2. Sample information of microarray datasets**

| **ID** | **Age** | **Sex** | **Evolution time** | **Condition** |
| --- | --- | --- | --- | --- |
| GSM1275185 | 33 | female | 6.63 | CD |
| GSM1275186 | 32 | female | 6.29 | CD |
| GSM1275187 | 36 | female | 7.95 | CD |
| GSM1275188 | 34 | male | 8.88 | CD |
| GSM1275189 | 33 | male | 5.65 | CD |
| GSM1275190 | 67 | male | 3.09 | CD |
| GSM1275191 | 21 | male | 0.69 | CD |
| GSM1275192 | 47 | male | 0.94 | CD |
| GSM1275193 | 34 | male | 1.36 | CD |
| GSM1275194 | 68 | male | 18.77 | CD |
| GSM948571 | 40 | female | 13 | UC |
| GSM948573 | 34 | male | 7 | UC |
| GSM948575 | 39 | male | 3 | UC |
| GSM948577 | 41 | female | 7 | UC |
| GSM948579 | 47 | female | 1 | UC |
| GSM948581 | 34 | female | 13 | UC |
| GSM948583 | 43 | female | 7 | UC |
| GSM948585 | 58 | male | 10 | UC |
| GSM948586 | 36 | female | 0 | UC |
| GSM948587 | 30 | female | 3 | UC |
| GSM948588 | 41 | female | 15 | UC |
| GSM948589 | 34 | female | 14 | UC |
| GSM948590 | 62 | female | 4 | UC |
| GSM948591 | 51 | male | 2 | UC |
| GSM948592 | 46 | female | 0 | UC |
| GSM1275212 | 51 | male | 0 | Control |
| GSM1275213 | 49 | male | 0 | Control |
| GSM1275216 | 44 | female | 0 | Control |
| GSM1275220 | 55 | male | 0 | Control |
| GSM1275222 | 50 | male | 0 | Control |
| GSM948550 | 20 | male | 0 | Control |
| GSM948551 | 30 | male | 0 | Control |
| GSM948552 | 41 | male | 0 | Control |
| GSM948555 | 55 | female | 0 | Control |
| GSM948556 | 36 | female | 0 | Control |
| GSM948558 | 22 | female | 0 | Control |
| GSM948559 | 38 | female | 0 | Control |
| GSM948560 | 50 | male | 0 | Control |
