# Supplementary material for: IBD Subtype-Regulators IFNG and GBP5 Identified by Causal Inference Drive More Intense Innate Immunity and Inflammatory Responses in CD Than Those in UC
Source: Front Pharmacol. 2022 Apr 6;13:869200. doi: 10.3389/fphar.2022.869200 (PMC9020454; doi:10.3389/fphar.2022.869200)
Supplement: Supplementary file 10 [file Table5.DOCX]

**Supplementary Table 5. Nucleotide sequences of primers used in qRT-PCR**

| **Gene** | **Primers (5’ to 3’)** | **Length (bp)** |
| --- | --- | --- |
| *IFNG* | (Forward) ACTGACTTGAATGTCCAACGCA | 22 |
|  | (Reverse) ATCTGACTCCTTTTTCGCTTCC | 22 |
| *GBP5* | (Forward) CCTGATGATGAGCTAGAGCCTG | 22 |
|  | (Reverse) GCACCAGGTTCTTTAGACGAGA | 22 |
| *NLRP3* | (Forward) GATCTTCGCTGCGATCAACAG | 21 |
|  | (Reverse) CGTGCATTATCTGAACCCCAC | 21 |
