# Supplementary material for: IBD Subtype-Regulators IFNG and GBP5 Identified by Causal Inference Drive More Intense Innate Immunity and Inflammatory Responses in CD Than Those in UC
Source: Front Pharmacol. 2022 Apr 6;13:869200. doi: 10.3389/fphar.2022.869200 (PMC9020454; doi:10.3389/fphar.2022.869200)
Supplement: Supplementary file 11 [file Table3.DOCX]

**Supplementary Table 3.** **Sample characteristics of the qPCR validation cohort**

| **Factor** | **Crohn's disease (CD)** | **Ulcerative colitis (UC)** | **Control** |
| --- | --- | --- | --- |
| Sample size | 36 | 13 | 33 |
| Gender (Male/Female) | 24/12 | 7/6 | 20/13 |
| Age（Year） | 27.9±9.2 | 35.1±15.3 | 45.6±10.6 |
