# Supplementary material for: IBD Subtype-Regulators IFNG and GBP5 Identified by Causal Inference Drive More Intense Innate Immunity and Inflammatory Responses in CD Than Those in UC
Source: Front Pharmacol. 2022 Apr 6;13:869200. doi: 10.3389/fphar.2022.869200 (PMC9020454; doi:10.3389/fphar.2022.869200)
Supplement: Supplementary file 12 [file Table4.DOCX]

**Supplementary Table 4.** **Sample information details of the qPCR validation cohort**

| **Sample ID** | **Age** | **Gender** | **Height** | **Weight** | **BMI** | **Diagnosis** |
| --- | --- | --- | --- | --- | --- | --- |
| F1 | 15 | Male | 156 | 36 | 14.8 | CD |
| F2 | 19 | Female | 149 | 40 | 18.0 | CD |
| F3 | 41 | Male | 168 | 69 | 24.4 | Control |
| F4 | 39 | Male | 176 | 67 | 21.6 | CD |
| F5 | 28 | Female | 160 | 41 | 16.0 | UC |
| F6 | 15 | Male | 179 | 53 | 16.5 | CD |
| F7 | 54 | Male | 168 | 70 | 24.8 | Control |
| F8 | 37 | Female | 168 | 50 | 17.7 | Control |
| F9 | 18 | Male | 175 | 58 | 18.9 | CD |
| F10 | 34 | Female | 163 | 46 | 17.3 | UC |
| F11 | 27 | Male | 160 | 57 | 22.3 | CD |
| F12 | 28 | Male | 170 | 53 | 18.3 | CD |
| F13 | 38 | Female | 155 | 58 | 24.1 | Control |
| F14 | 40 | Male | 158 | 62 | 24.8 | CD |
| F15 | 60 | Male | 165 | 70 | 25.7 | Control |
| F16 | 54 | Female | 150 | 58 | 25.8 | Control |
| F17 | 29 | Male | 175 | 67 | 21.9 | UC |
| F18 | 19 | Male | 175 | 56 | 18.3 | CD |
| F19 | 35 | Male | 165 | 65 | 23.9 | Control |
| F20 | 39 | Female | 148 | 88 | 40.2 | Control |
| F21 | 16 | Female | 156 | 45 | 18.5 | CD |
| F22 | 15 | Male | 170 | 51 | 17.6 | Control |
| F23 | 38 | Female | 164 | 53 | 19.7 | CD |
| F24 | 30 | Male | 163 | 44.5 | 16.7 | CD |
| F25 | 47 | Female | 160 | 48 | 18.8 | Control |
| F26 | 30 | Male | 163 | 56 | 21.1 | CD |
| F27 | 33 | Female | 161 | 50.5 | 19.5 | CD |
| F28 | 25 | Male | 176 | 57 | 18.4 | CD |
| F29 | 62 | Female | 147 | 60 | 27.8 | Control |
| F30 | 15 | Male | 174 | 50 | 16.5 | CD |
| F31 | 17 | Male | 164 | 44 | 16.4 | CD |
| F32 | 45 | Female | 150 | 46.5 | 20.7 | CD |
| F33 | 48 | Male | 173 | 60 | 20.0 | Control |
| F34 | 30 | Male | 163 | 52 | 19.6 | CD |
| F35 | 26 | Female | 158 | 40 | 16.0 | CD |
| F36 | 32 | Female | 165 | 49 | 18.0 | UC |
| F37 | 49 | Male | 170 | 80 | 27.7 | Control |
| F38 | 56 | Male | 175 | 65 | 21.2 | Control |
| F39 | 36 | Female | 160 | 54 | 21.1 | CD |
| F40 | 28 | Male | 170 | 63 | 21.8 | CD |
| F41 | 19 | Male | 175 | 71 | 23.2 | UC |
| F42 | 24 | Male | 185 | 64 | 18.7 | CD |
| F43 | 26 | Male | 170 | 42.5 | 14.7 | CD |
| F44 | 34 | Male | 166 | 45 | 16.3 | CD |
| F45 | 43 | Male | 172 | 78.5 | 26.5 | Control |
| F46 | 46 | Male | 165 | 61 | 22.4 | Control |
| F47 | 27 | Male | 167 | 42 | 15.1 | CD |
| F48 | 52 | Female | 150 | 45 | 20.0 | CD |
| F49 | 18 | Male | 173 | 50 | 16.7 | CD |
| F50 | 58 | Male | 168 | 72.5 | 25.7 | Control |
| F51 | 43 | Male | 173 | 71 | 23.7 | Control |
| F52 | 57 | Male | 168 | 51 | 18.1 | UC |
| F53 | 47 | Female | 160 | 53 | 20.7 | Control |
| F54 | 18 | Male | 173 | 45.5 | 15.2 | UC |
| F55 | 26 | Female | 160 | 40 | 15.6 | CD |
| F56 | 46 | Male | 163 | 60 | 22.6 | Control |
| F57 | 42 | Male | 170 | 60 | 20.8 | Control |
| F58 | 51 | Male | 166 | 68 | 24.7 | Control |
| F59 | 47 | Female | 160 | 63 | 24.6 | Control |
| F60 | 69 | Male | 173 | 80 | 26.7 | Control |
| F61 | 54 | Female | 160 | 55 | 21.5 | Control |
| F62 | 55 | Male | 173 | 66 | 22.1 | Control |
| F63 | 23 | Female | 165 | 58 | 21.3 | CD |
| F64 | 37 | Male | 170 | 73 | 25.3 | Control |
| F65 | 44 | Male | 165 | 76.5 | 28.1 | Control |
| F66 | 39 | Male | 169 | 74 | 25.9 | Control |
| F67 | 29 | Female | 161 | 54 | 20.8 | Control |
| F68 | 38 | Male | 178 | 57 | 18.0 | CD |
| F69 | 33 | Female | 152 | 50 | 21.6 | Control |
| F70 | 51 | Female | 160 | 51 | 19.9 | Control |
| F71 | 15 | Male | 170 | 78 | 27.0 | UC |
| F72 | 33 | Male | 168 | 49 | 17.4 | CD |
| F73 | 25 | Male | 176 | 68 | 22.0 | CD |
| F74 | 49 | Female | 150 | 62 | 27.6 | UC |
| F75 | 56 | Female | 165 | 63 | 23.1 | UC |
| F76 | 21 | Male | 173 | 51 | 17.0 | UC |
| F77 | 39 | Male | 168 | 56.5 | 20.0 | CD |
| F78 | 44 | Female | 163 | 53 | 19.9 | UC |
| F79 | 34 | Female | 155 | 41 | 17.1 | CD |
| F80 | 36 | Female | 160 | 62 | 24.2 | Control |
| F81 | 54 | Male | 170 | 77.5 | 26.8 | UC |
| F82 | 19 | Female | 140 | 36 | 18.4 | CD |
