# Supplementary material for: IBD Subtype-Regulators IFNG and GBP5 Identified by Causal Inference Drive More Intense Innate Immunity and Inflammatory Responses in CD Than Those in UC
Source: Front Pharmacol. 2022 Apr 6;13:869200. doi: 10.3389/fphar.2022.869200 (PMC9020454; doi:10.3389/fphar.2022.869200)
Supplement: Supplementary file 14 [file Table6.DOCX]

**Supplementary Table 6. Differentially expressed pathway between CD and UC**

| **KEGG ID** | **Description** | ***P* value** |
| --- | --- | --- |
| hsa04621 | NOD-like receptor signaling pathway | 0.02 |
| hsa05150 | Staphylococcus aureus infection | 0.024 |
| hsa04657 | IL-17 signaling pathway | 0.025 |
| hsa05323 | Rheumatoid arthritis | 0.025 |
| hsa04668 | TNF signaling pathway | 0.027 |
| hsa05202 | Transcriptional misregulation in cancer | 0.034 |
| hsa04062 | Chemokine signaling pathway | 0.05 |

**P*-value was sorted by ascending.
